# Supplementary material for: Antimicrobial resistant enteric bacteria are widely distributed amongst people, animals and the environment in Tanzania
Source: Nat Commun. 2020 Jan 13;11:228. doi: 10.1038/s41467-019-13995-5 (PMC6957491; doi:10.1038/s41467-019-13995-5)
Supplement: Supplementary file 1 — Supplementary Information [file 41467_2019_13995_MOESM1_ESM.pdf]

Supporting Information

Title: Antimicrobial resistant enteric bacteria are widely distributed amongst people animals and the environment in Tanzania

Authors: Murugan Subbiah[1,†], Mark A Caudell[1,2,†,*], Colette Mair[3,†], Margaret A. Davis[1], Louise Matthews[3], Robert J.Quinlan[1,4], Marsha B. Quinlan[1,4] Beatus Lyimo[5], Joram Buza[5], Julius Keyyu[6] and Douglas R. Call[1,5]

Affiliations: [1] Paul G. Allen School for Global Animal Health, Washington State University, Pullman, WA [2] Food and Agriculture Organization of the United Nations, Nairobi, Kenya, [3]Boyd Orr Centre for Population and Ecosystem Health, Institute of Biodiversity, Animal Health and Comparative Medicine, University of Glasgow, Glasgow, UK, [4] Department of Anthropology, Washington State University, Pullman, WA, [5] Nelson Mandela African Institution of Science and Technology, Arusha, Tanzania, [6] Tanzania Wildlife Research Institute, Arusha, Tanzania

Supplementary Figure 1. **Minimum spanning tree for *E. coli* MLVA haplotypes obtained from animals and people.**

A. People, dogs, chicken vs livestock (red) | B. People (red) vs dogs and chicken | C. People vs livestock (red)

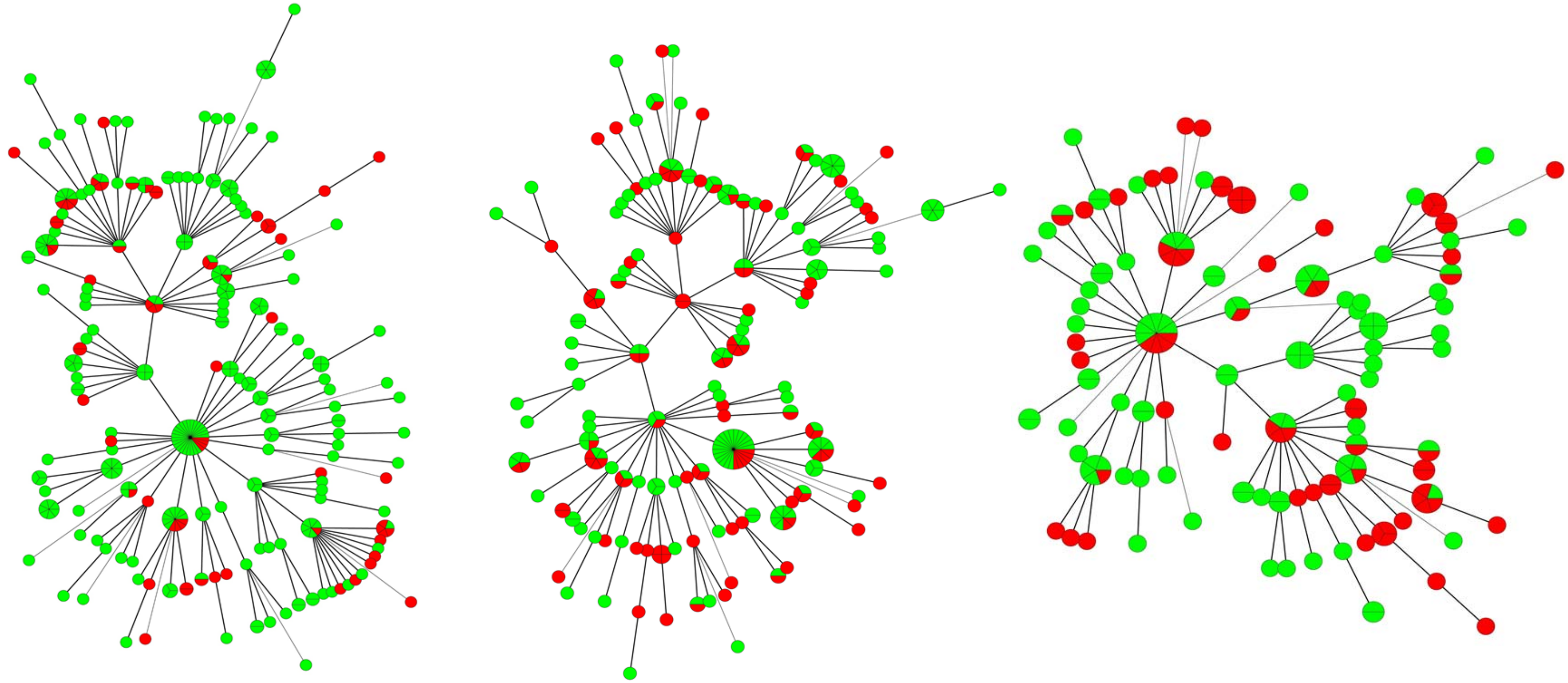

Each circle or pie-slice represent a single *E. coli* isolate. Most of the isolates are differed by single locus (solid lines) and form a single clonal complex.

Supplementary Figure 2. Phylogenetic tree derived from 82 *E. coli* isolates collected from 8 selected Maasai households.

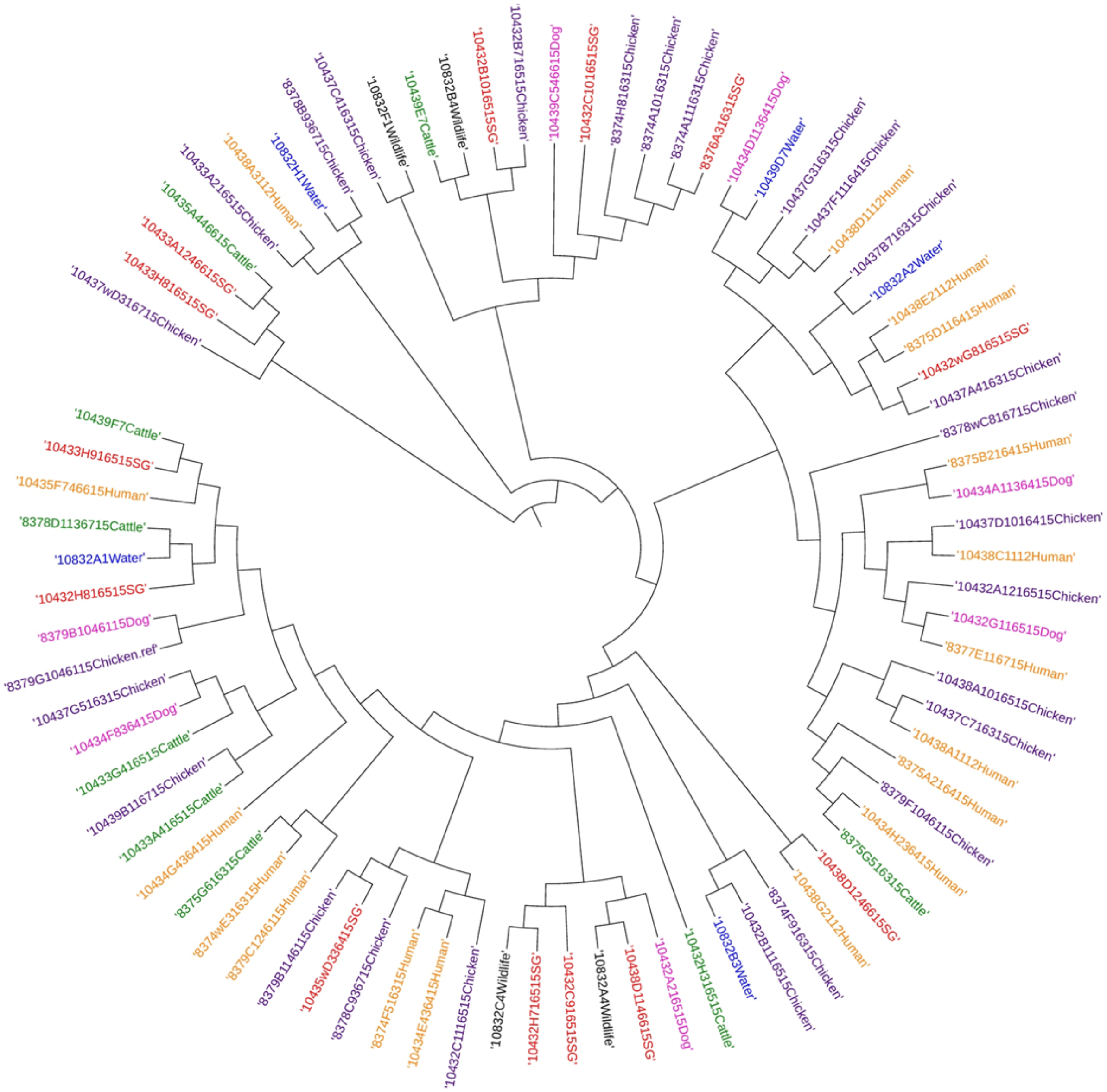

No clustering of isolates was noticed based on core-genome-based (S6) trees. Labels show barcode id, house id, year of collection and host species name (e.g. 10435D546615Human; 10435D5 – barcode id, 466 – household id, 15 – year of collection, 2015 and Human – host species).

Supplementary Figure 3. Prevalence of antimicrobial resistant E. coli.

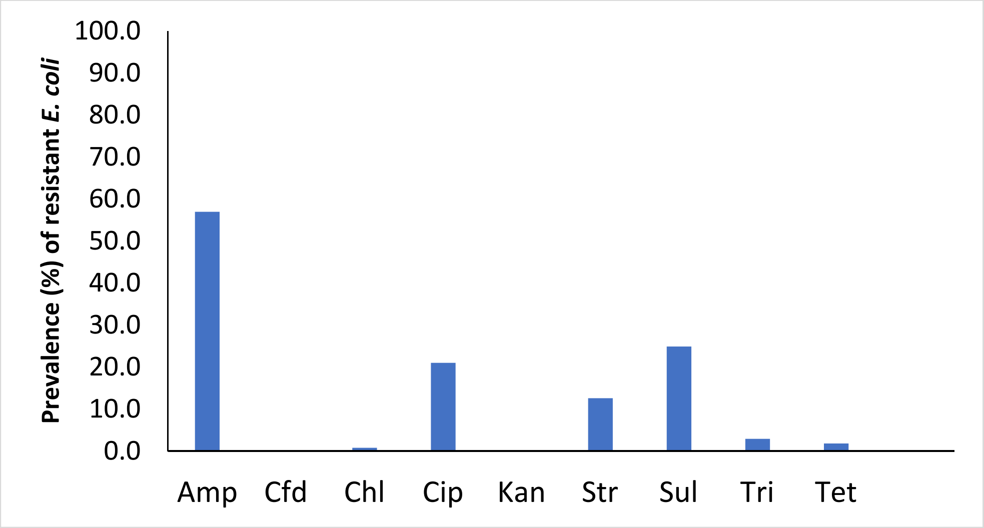

E. coli isolated from a municipal waste sample (Pullman, WA, N = 381 isolates). Antibiotics included amp (ampicillin), cfd (ceftazidime), chm (chloramphenicol), cip, (ciprofloxacin) and kan (kanamycin), str, (streptomycin), sul (sulfamethoxazole), tri (trimethoprim), and tet (tetracycline).

Supplementary Table 1. Number of households surveyed by year, village/ward, season, and ethnicity.

Ethnicity (number of households)	Village/Ward	Season	Year(s)
Maasai (N=163)	Monduli	Wet & Dry	2013/15
	Loibor Siret	Dry	2013/15
	Terat	Wet	2013
	Nadonjukin	Wet & Dry	2013/14/15
	Loliondo	Dry	2013
	Komolo	Dry	2015
Arusha (N=97)	Aremeru ward	Wet & Dry	2013/15
	Loroi	Dry	2015
	Meliot	Dry	2015
Chagga (N=94)	Masaera kati	Wet & Dry	2014
	Masaera juu	Dry	2014
	Mamsera chini	Dry	2014
	Mamsera juu	Dry	2014

Supplementary Table 2. Primers used for multi-locus variable number of tandem repeats analysis [23].

Primer ID	Primer sequences[#]	Tag color*	Product size[$]
CVN001-F	*6FAM*-AAC CGG CTG GGG CGA ATC C	Yellow	
CVN001-R	GGC GGC GGT GTC AGC AAA TC		445-484
CVN004-F	*PET*-GCT GCG GCC TGA AGA AGA	Green	
CVN004-R	CCC GGC AGG CGA AGC ATT GT		322, 403
CVN015-F	*TET*-TAG GCA TAG CGC ACA GAC AGA TAA	Blue	
CVN015-R	GTA CCG CCG AAC TTC AAC ACT C		220, 237, 931
CVN014-F	*6FAM*-TCC CCG CAA TCA GCA AAC AAA GA	Blue	123-159
CVN014-R	GCA GCG GGA CAA CGG AAG C		
CVN016-F	*NED*-GCA ATC ACC GCC GCA ATC TGT T	Yellow	461-611
CVN016-R	CGC CGC CGA AGC AAA TCT C		

*These colors were used to identify the fluorophores used in the primers; 6FAM (yellow), PET (green), TET (blue).

[$] range of product sizes found with the current study.

[#] Multiplex reaction mix – 1 contains CVN001, CVN004 and CVN015 primers; multiplex mix 2 contains CVN014 and CVN016
