# Supplementary material for: Antimicrobial resistant enteric bacteria are widely distributed amongst people, animals and the environment in Tanzania
Source: Nat Commun. 2020 Jan 13;11:228. doi: 10.1038/s41467-019-13995-5 (PMC6957491; doi:10.1038/s41467-019-13995-5)
Supplement: Supplementary file 3 — Reporting Summary [file 41467_2019_13995_MOESM3_ESM.pdf]

natureresearch

Corresponding author(s): Mark Caudell

Last updated by author(s): Nov 6, 2019

Reporting Summary

Nature Research wishes to improve the reproducibility of the work that we publish. This form provides structure for consistency and transparency in reporting. For further information on Nature Research policies, see Authors & Referees and the Editorial Policy Checklist .

Statistics

For all statistical analyses, confirm that the following items are present in the figure legend, table legend, main text, or Methods section.

n/a	Confirmed	
☐	☒	The exact sample size (*n*) for each experimental group/condition, given as a discrete number and unit of measurement
☐	☒	A statement on whether measurements were taken from distinct samples or whether the same sample was measured repeatedly
☐	☒	The statistical test(s) used AND whether they are one- or two-sided<br>*Only common tests should be described solely by name; describe more complex techniques in the Methods section.*
☐	☒	A description of all covariates tested
☐	☒	A description of any assumptions or corrections, such as tests of normality and adjustment for multiple comparisons
☐	☒	A full description of the statistical parameters including central tendency (e.g. means) or other basic estimates (e.g. regression coefficient) AND variation (e.g. standard deviation) or associated estimates of uncertainty (e.g. confidence intervals)
☐	☒	For null hypothesis testing, the test statistic (e.g. *F*, *t*, *r*) with confidence intervals, effect sizes, degrees of freedom and *P* value noted<br>*Give P values as exact values whenever suitable.*
☒	☐	For Bayesian analysis, information on the choice of priors and Markov chain Monte Carlo settings
☐	☒	For hierarchical and complex designs, identification of the appropriate level for tests and full reporting of outcomes
☐	☒	Estimates of effect sizes (e.g. Cohen's *d*, Pearson's *r*), indicating how they were calculated

Our web collection on statistics for biologists contains articles on many of the points above.

Software and code

Policy information about availability of computer code

Data collection	Survey Gizmo was used to program and collect the socioeconomic survey.
Data analysis	Bionumerics version 6.6 was used to create MLVA cluster images; Bioinformatics utilities from Center for Genomic Epidemiology was used to analyze whole genome sequences including MLST analyses; MEGA7 program was used to develop mlst phylogenetic tree; Harvest suite was used to generate phylogenetic tree using whole genome comparison; iTOL online platform was used to manage phylogenetic trees. Stata, version 15.1 was used for mixed effects regression modeling (package melogit). R, version 3.5, was used to generate prevalence figures (Figure 2 and Figure 3). ArcGIS Pro, version 2.4.2, was used to compile the study map (Figure 1).

For manuscripts utilizing custom algorithms or software that are central to the research but not yet described in published literature, software must be made available to editors/reviewers. We strongly encourage code deposition in a community repository (e.g. GitHub). See the Nature Research guidelines for submitting code & software for further information.

Data

Policy information about availability of data

All manuscripts must include a data availability statement. This statement should provide the following information, where applicable:
- Accession codes, unique identifiers, or web links for publicly available datasets
- A list of figures that have associated raw data
- A description of any restrictions on data availability

Sequence data that support the findings of this study have been deposited in GenBank at National Center for Biotechnology Information [repository name “BioProject ID PRJNA578301; Genome submission SUB6444306”] with the accession codes SAMN13068707 through SAMN1068790. The socioeconomic data that support findings of this survey are available at figshare with identifiers doi: 10.6084/m9.figshare.10185077.

Field-specific reporting

Please select the one below that is the best fit for your research. If you are not sure, read the appropriate sections before making your selection.

☐ Life sciences ☒ Behavioural & social sciences ☐ Ecological, evolutionary & environmental sciences

For a reference copy of the document with all sections, see nature.com/documents/nr-reporting-summary-flat.pdf

Behavioural & social sciences study design

All studies must disclose on these points even when the disclosure is negative.

Study description	The study was a quantitative and cross-sectional. The socioeconomic survey used for quantitative data collection was developed through qualitative assessments, primarily focus group discussions and key information interviews.
Research sample	The research sample was composed of three ethnic groups in northern Tanzania, the Arusha, the Chagga, and the Maasai. For the final sample, 164 respondents were Maasai, 97 were Chagga and 94 were Arusha. Across the sample, average age was 46 years with 61% of respondents (216 respondents) being males and the remaining females (138). Ninety percent of the sample was married (321), 3% were widowed (10), 0.56% were divorced (2) and 6% (21) had never married. The average number of children was apprx. 7. The average education level was "some primary school". The Maasai, Chagga, and Arusha communities were sampled because they vary considerably across social dimensions that have been routinely proposed as potential risk factors for antimicrobial resistance, including education and income levels, antimicrobial use and access, animal husbandry practices, proximity to urban environments, and hygiene and sanitation practices. However, the Chagga, Maasai, and Arusha also live in close proximity to each other, such that households buy and sell livestock and crops at the same markets, visit the same hospitals and pharmacies, and deal with the same regulations and policies that guide human and animal health care in Tanzania. The study was not representative of Arusha, Chagga, or Maasai ethnic groups.
Sampling strategy	For the socioeconomic data, sampling was conducted at the village and household levels. At the village level, selection was purposive using the criteria of 1) main ethnic composition of the village, 2) distance to wildlife areas and urban areas, 3) whether other research projects were being conducted in the area, and 4) in consultation with enumerators and village chairmen. At the household level, selection was random, with households randomly selected from census lists provided by local district offices. Power calculations were based upon the likely frequency of observing resistant bacteria at the household level (assumed 50%) with an acceptable margin of error (assumed 10%) and a design effect of 2. Assuming three clusters and a 95% confidence interval, it was calculated that 64 households per cluster were needed. The calculation was based on StatCalc-Sample Size and Power Calculator published by the CDC as the Epi Info software package (version 7.2.3.1). While our target was 64 households per ethnic group, we strove to sample 30 per village given a desire to examine between-village differences in risk factors for AMR.
Data collection	Data were collected using tablets uploaded with the Survey Gizmo application. Individuals present at interviews were enumerators, participants, and sometimes a village chairman who introduced the research team to the household. Researchers were not blind to study hypotheses during data collection. Fecal data were collected using whirlpaks and gloves. Water samples were collected in 50ml tubes. The research team collected all fecal samples.
Timing	Socioeconomic and fecal data collection were conducted between March 2012-July 2015. Data collection was not continuous. Collection was focused on the summer months May, June, July, August, as this is the dry season in the study area which facilitated traveling to and from sampled villages
Data exclusions	No data were excluded from the analysis
Non-participation	No participants dropped out of the study as it was cross-sectional. A number of randomly selected households refused to participate across villages. In almost all cases, this was due to household heads not being available during the study period. In a limited number of cases, individuals refused to participate due to health reasons
Randomization	Participants were not allocated into experimental groups

Reporting for specific materials, systems and methods

We require information from authors about some types of materials, experimental systems and methods used in many studies. Here, indicate whether each material, system or method listed is relevant to your study. If you are not sure if a list item applies to your research, read the appropriate section before selecting a response.

Materials & experimental systems

n/a	Involved in the study
☒	☐ Antibodies
☒	☐ Eukaryotic cell lines
☒	☐ Palaeontology
☐	☒ Animals and other organisms
☐	☒ Human research participants
☒	☐ Clinical data

Methods

n/a	Involved in the study
☒	☐ ChIP-seq
☒	☐ Flow cytometry
☒	☐ MRI-based neuroimaging

Animals and other organisms

Policy information about studies involving animals; ARRIVE guidelines recommended for reporting animal research

Laboratory animals	We did not use laboratory animals
Wild animals	Voided fecal samples were collected from wildlife, but no animals were handled or otherwise manipulated or disturbed.
Field-collected samples	Voided feces
Ethics oversight	A research permit for collecting voided wildlife fecal samples were issued by the Tanzania Wildlife Research Institute (No. 2015—127-ER-2012-51)

Note that full information on the approval of the study protocol must also be provided in the manuscript.

Human research participants

Policy information about studies involving human research participants

Population characteristics	See above
Recruitment	Lists of households in villages were compiled from data at district offices. These lists were consulted to randomly select households (up to 50 per village). Household heads were contacted by village chairmen to assess willingness to participate. Chairmen accompanied the research team to randomly selected households that were willing to participate.
Ethics oversight	The study protocols were reviewed and approved by the Washington State University (IRB #12355) and Tanzania National Institute for Medical Research institutional review boards, and a research permit was issued by the Tanzania Commission for Science and Technology (permit 2012-151).

Note that full information on the approval of the study protocol must also be provided in the manuscript.
