# Supplementary material for: Antimicrobial resistant enteric bacteria are widely distributed amongst people, animals and the environment in Tanzania
Source: Nat Commun. 2020 Jan 13;11:228. doi: 10.1038/s41467-019-13995-5 (PMC6957491; doi:10.1038/s41467-019-13995-5)
Supplement: Supplementary file 4 — Description of Additional Supplementary Files [file 41467_2019_13995_MOESM4_ESM.pdf]

**Description of Additional Supplementary Files**

File Name: Supplementary Data 1
Description: **Census of antibiotic resistance genes.** Genes identified by ResFinder software from 816 E. coli whole-genome sequences from this study: cattle (n = 132), sheep and goats (n = 162), chicken (n = 209), dog (n = 107), people (n = 165), wildlife (n = 18), and water (n = 23).

File Name: Supplementary Data 2
Description: **Livestock, all groups. Odds** ratios (95% confidence intervals) for mixed-effects multivariate logistic regression models for bacterial isolates collected from livestock and pooled across ethnic groups livestock. See Table 5 in manuscript for variable descriptions. MDR is defined as resistance to three or more antibiotics.

File Name: Supplementary Data 3
Description: **Arusha livestock.** Odds ratios (95% confidence intervals) for mixed-effects multivariate logistic regression models for bacterial isolates collected from Arusha. See Table 5 in manuscript for variable descriptions. MDR is defined as resistance to three or more antibiotics.

File Name: Supplementary Data 4
Description: **Chagga livestock.** Odds ratios (95% confidence intervals) for mixed-effects multivariate logistic regression models for bacterial isolates collected from Chagga livestock. See Table 5 in manuscript for variable descriptions. MDR is defined as resistance to three or more antibiotics.

File Name: Supplementary Data 5
Description: **Maasai livestock.** Odds ratios (95% confidence intervals) for mixed-effects multivariate logistic regression models for bacterial isolates collected from Maasai livestock. See Table5 in manuscript for variable descriptions. MDR is defined as resistance to three or more antibiotics.

File Name: Supplementary Data 6
Description: **Chicken, all groups.** Odds ratios (95% confidence intervals) for mixed-effects multivariate logistic regression models for bacterial isolates collected from chicken and pooled across ethnic groups. See Table 5 in manuscript for variable descriptions. MDR is defined as resistance to three or more antibiotics.

File Name: Supplementary Data 7
Description: **Dogs, all groups.** Odds ratios (95% confidence intervals) for mixed-effects multivariate logistic regression models for bacterial isolates collected from chicken and pooled across ethnic groups. See Table 5 in manuscript for variable descriptions. MDR is defined as resistance to three or more antibiotics.

File Name: Supplementary Data 8
Description: **Descriptive statistics of variables entered into multivariate models by ethnic group.**

File Name: Supplementary Data 9
Description: **Fit Indices for mixed-effects logistic models.** McKelvey and Zavoina Pseudo r2 and Intraclass Correlations.
